# What Macromolecular Crowding Can Do to a Protein

**DOI:** 10.3390/ijms151223090

**Published:** 2014-12-12

**Authors:** Irina M. Kuznetsova, Konstantin K. Turoverov, Vladimir N. Uversky

**Affiliations:** 1 Laboratory of Structural Dynamics, Stability and Folding of Proteins, Institute of Cytology, Russian Academy of Sciences, 4 Tikhoretsky Ave., St. Petersburg 194064, Russia; E-Mails: imk@mail.cytspb.rssi.ru (I.M.K.); kkt@incras.ru (K.K.T.); 2 Department of Biophysics, St. Petersburg State Polytechnical University, 29 Polytechnicheskaya St., St. Petersburg 195251, Russia; 3 Department of Molecular Medicine and USF Health Byrd Alzheimerʼs Research Institute, Morsani College of Medicine, University of South Florida, 12901 Bruce B. Downs Blvd. MDC07, Tampa, FL 33620, USA; 4 Institute for Biological Instrumentation, Russian Academy of Sciences, 4 Institutskaya St., Pushchino, Moscow 142290, Russia; 5 Biology Department, Faculty of Science, King Abdulaziz University, P.O. Box 80203, Jeddah 21589, Saudi Arabia

**Keywords:** macromolecular crowding, excluded volume, protein structure, protein folding, protein function, protein-protein interaction, intrinsically disordered protein, protein aggregation

## Abstract

The intracellular environment represents an extremely crowded milieu, with a limited amount of free water and an almost complete lack of unoccupied space. Obviously, slightly salted aqueous solutions containing low concentrations of a biomolecule of interest are too simplistic to mimic the “real life” situation, where the biomolecule of interest scrambles and wades through the tightly packed crowd. In laboratory practice, such macromolecular crowding is typically mimicked by concentrated solutions of various polymers that serve as model “crowding agents”. Studies under these conditions revealed that macromolecular crowding might affect protein structure, folding, shape, conformational stability, binding of small molecules, enzymatic activity, protein-protein interactions, protein-nucleic acid interactions, and pathological aggregation. The goal of this review is to systematically analyze currently available experimental data on the variety of effects of macromolecular crowding on a protein molecule. The review covers more than 320 papers and therefore represents one of the most comprehensive compendia of the current knowledge in this exciting area.

## 1. Introduction

**Figure 1:**
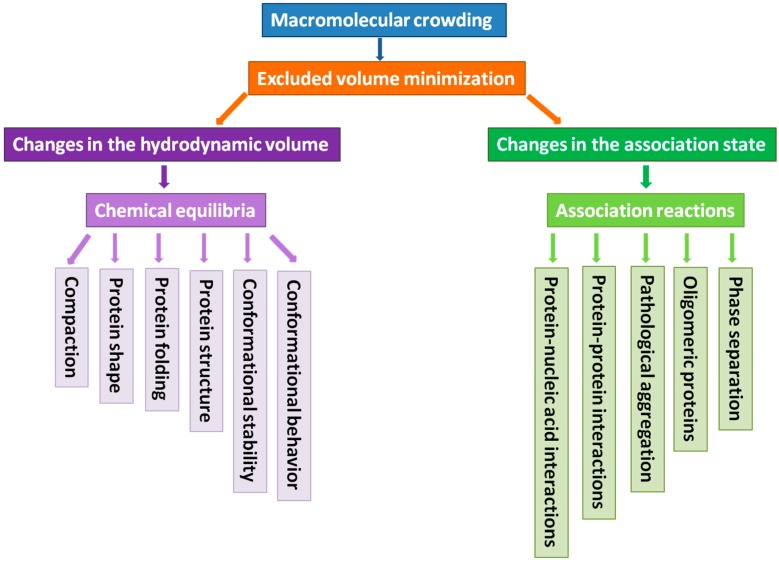
Schematic representation of the potential effects of excluded volume on the behavior of proteins in crowded milieu.

The intracellular environment is extremely crowded. Estimates show that the concentration of biological macromolecules (proteins, nucleic acids, ribonucleoproteins, polysaccharides, *etc.*) inside cells is in the range of 80–400 mg/mL [1,2,3]. This corresponds to a volume occupancy of 5%–40% [4] and creates a crowded medium, with considerably restricted amounts of free water [1,5,6,7,8,9]. Such natural intracellular media, being filled with billions of protein molecules [10] and a myriad of DNA, RNA, and polysaccharide molecules are known as “crowded” rather than “concentrated” environments, as, in general, no individual macromolecular species may be present at high concentration [7,11]. Obviously, the average spacing between macromolecules in such crowded milieu can be much smaller than the size of the macromolecules themselves [12]. Furthermore, the volume occupied by solutes is unavailable to other molecules because two molecules cannot be in the same place at the same time. As a result, any reactions that depend on available volume can be affected by macromolecular crowding effects [5,13], and the thermodynamic consequences of the unavailable volume are called excluded volume effects [5,14]. In other words, the fact that two molecules cannot occupy the same space in solution, and that steric hindrance or impediment of a macromolecule is expected to exclude other molecules from its neighborhood give rise to the excluded volume phenomenon [15]. In solutions with increasing concentrations of such particles, the number of ways that can be used to place added molecules is progressively limited since the volume of solution available to the new molecules is progressively restricted to the part of space from which they are not excluded [15]. The consequence of this phenomenon is decreased randomness of the particle distribution in the concentrated solutions leading to the noticeable decrease in the entropy of the crowded solution. Such entropy decrease increases the free energy of the solute and thereby produces an increase in the thermodynamic activity of solute, and therefore is expected to affect various processes determined by the activity [15]. Importantly for this review, the mentioned excluded volume effects do not only influence the thermodynamic activity of the concentrated solute itself but also affects any other solute present in the solution at low concentrations [15]. Therefore, the behavior of a molecule of interest in the non-ideal crowed solutions is expected to be different from the behavior of this molecule in the diluted solutions [15]. In agreement with these considerations, using various *in vitro* systems with model crowded conditions, volume exclusion was shown to have large effects on conformational stability and structural properties of biological macromolecules [11,16,17,18]. Also, macromolecular crowding may affect various biological equilibria, such as protein folding, binding of small molecules, enzymatic activity, protein-protein interactions [11,19], pathological protein aggregation, and extent of amyloid formation [20,21,22]. This review represents an analysis of available experimental data on what macromolecular crowding can do to a protein molecule. Figure 1 provides an oversimplified view of macromolecular crowding effects on a protein molecule. It shows that the major mechanism by which macromolecular crowding is expected to affect proteins is related to the minimization of excluded volume. This tendency for the excluded volume minimization in crowded environments is driven by the need of a system to maximize its entropy. The excluded volume can be minimized either by changes in the hydrodynamic volume of a protein or via changes in its oligomerization/association state. Changes in hydrodynamic volume are promoted by modulation of the chemical equilibria that would affect protein folding, structure, shape, compaction, conformational stability, and conformational equilibrium. Changes in the association state are controlled by modulating the association reactions and would have effects on protein-protein interactions, protein–nucleic acid interactions, protein oligomerization, pathologic aggregation, and phase separations.

Although it is assumed that the behavior of a protein in a crowded environment can be reliably explained in terms of the hard non-specific steric interactions between the protein and crowding agents (which constitute the basis for the excluded volume effect), it is almost impossible to find a completely inert polymer. Therefore, in some systems binding of the protein of interest to the crowding agent can be a major problem. This means that special precautions should be taken in performing the crowding experiments and in interpreting the retrieved data. To this end, the lack of significant interaction between the protein and crowder should be demonstrated and/or experiments should be analyzed in media with different crowding agents.

## 2. What Can Macromolecular Crowding Do to a Protein

### 2.1. How to Model Macromolecular Crowding?

The real “life” of a protein takes place within a highly packed space, a kind of very thick soup, which is hardly mimicked by the so-called “physiological conditions” used in the vast majority of *in vitro* experiments where studies are conducted under the relatively ideal thermodynamic conditions of low protein and moderate salt concentrations. However, there is an increasing appreciation of this shortcoming and the view that macromolecular crowding is an important yet neglected variable in biochemical studies has gained attention [5,8]. As a first approximation, effects of macromolecular crowding on biological macromolecules may be examined experimentally by using concentrated solutions of a model “crowding agent”, such as poly(ethylene glycol), dextran, Ficoll, or inert proteins [14,20]. The major reason for using concentrated solutions of such macromolecules is to mimic macromolecular crowding characterized by a high excluded volume; *i.e.*, the volume occupied by the crowder molecules that is unavailable to other biological macromolecules. In fact, as a result of the mutual impenetrability of solute molecules, steric repulsions are generated, and since the inert crowders occupy a significant volume fraction in the medium, such crowded environment is expected to place constraints on the active factors present in the microenvironment [8,14,23]. It is important to remember that based on the aforementioned considerations, the efficiency of macromolecular crowding is dependent on a ratio between the hydrodynamic dimensions (or occupied volumes) of the crowder and the test molecule, with the most effective conditions being those where the volumes occupied by the crowder and the test molecule are of similar size [23,24,25]. Therefore, the knowledge on the hydrodynamic radii of the crowding agents and the test molecules of interest is important for the rational selection of suitable crowding conditions [23]. Table 1 represents experimentally determined and calculated hydrodynamic radii of several of the most commonly used macromolecular crowders together with other experimentally determined parameters based on the effects of some of these crowding agents on accelerated collagen deposition [23].

Since the space available to a biological molecule in the cellular milieu is very limited, one can also use some experimental approaches to model such confined intracellular space. One of these approaches is the encapsulation of a studied protein in silica glass using the sol–gel techniques. Although the fraction of the total volume excluded by the silica matrix is less than the fractional volume occupied by macromolecules in living cell [16,17], the size of protein-occupied pores in these gels is believed to have the same order of magnitude as the diameter of protein, and the protein itself was suggested to dictate the pore size during the gelation process [26]. Proteins are not bound covalently to the silica matrix, but the matrix substantially impedes the rotational freedom of the protein [27]. The mild conditions of sol–gel glass encapsulation have proven to be compatible with the folded structure and function of several ordered proteins [28,29,30]. The optically transparent glass products may be analyzed by the majority of spectroscopic techniques used to monitor the structure of proteins in dilute solutions. This includes fluorescence [28,31] and circular dichroism (CD) [16,17]. Furthermore, the highly porous character of the glass allows easy exchange of the solvent that permeates the silica matrix, but the encapsulated macromolecules are unable to escape the glass under most solvent conditions [16]. Using this approach, solvent effects on the secondary structure of several ordered proteins have been analyzed by CD following encapsulation in the hydrated pores of a silica glass matrix by the sol–gel method [16,17].

**Table 1:** Hydrodynamic radii and related parameters of some crowding agents.

Macromolecular Crowders	Molecular Mass (kDa)	Hydrodynamic Radius (Å)	Effective Concentration ^a^	Fractional vol. Occupancy Ψ ^b^
Poly(ethylene glycol) PEG 2050	2	3.8 ^c^/11.3 ^d^		
Dextran sulfate 10 ^e^	10	<10		
Bovine pancreatic trypsin inhibitor (BPTI) ^f^	6.5	14.2		
PEG 4600 ^d^	4.6	17.9		
Ribonuclease A ^g^	13.7	19.3		
Lysozyme ^g^	14.3	20.0		
PEG 6000 ^d^	6.0	20.8		
PEG 8000 ^d^	8.0	24.5		
β-Lactoglobulin ^g^	36.8	27.1		
Hemoglobin ^g^	64.5	33.2		
Bovine serum albumin (BSA)^e^	66.3	33.9	80 mg/mL	18%
PEG 20000	20	34.5 ^h^/41.4 ^d^		
Ficoll 70 ^e^	70	40	37.5 mg/mL	17%
PEG 35000 ^d^	35	57.0		
Ficoll 400 ^e^	400	80	25 mg/mL	
Dextran 670 ^e^	670	210		
Poly(sodium 4-styrene sulfonate) (PSS) ^e^	200	220	50 µg/mL	0.7%
Dextran sulfate 500 ^e^	500	470	100 µg/mL	5.2%

^a^ The most effective concentrations were determined empirically in terms of accelerated collagen deposition [23]; ^b^ Fractional volume occupancy was calculated for some crowding agents based on their hydrodynamic radii and effective concentrations [23]; ^c^ Data are taken from [32]; ^d^ Data for PEGs of different molecular mass are calculated from their molecular masses using a known scaling law [33]: *R*_H_ = 0.145 × *M*_W_^0.571 ± 0.009^ Å. Note that PEGs in aqueous solutions are characterized by the ratio ρ = *R*_g_/*R*_H_ = 1.73, which is greater than the value estimated a Gaussian chain (ρ = 1.5) but is in a good agreement with theoretical predictions for swollen coils (ρ = 1.86) [33]; ^e^ Data are taken from [23]; ^f^ Calculated based on the empirical formula reported in [34]; ^g^ Data are taken from [34]; ^h^ Data for PEG 20000 are taken from [35].

### 2.2. Protein Function in Crowded Environments

#### 2.2.1. Ordered Monomeric Proteins

Since the efficiency of the macromolecular crowding depends on a ratio between the hydrodynamic dimensions of the inert crowder and the test molecule, and since the most effective conditions are achieved when these volumes are comparable [23,28,29], one would expect that the interaction of a substrate with enzyme would not be affected much by the excluded volume since the volume of a substrate is too small. However, when catalysis is accompanied by changes in the size or shape of an enzyme, or when the enzyme interacts with a large substrate, e.g., nucleic acids or proteins, then macromolecular crowding can affect an enzymatic reaction [15]. A few cases below provide support to this notion.

Zimmerman and Pheiffer analyzed the catalytic activity of the DNA ligases (EC 6.5.1.1) from rat liver nuclei and from *Escherichia coli* and showed that these enzymes were completely inactive under conventional assay conditions but actively catalyzed the blunt-end ligation of DNA in the presence of high concentrations of several various macromolecules, such as PEG 6000, Ficoll 70 and bovine serum albumin (BSA) [36]. Also, the similar activity of T4 DNA ligase on blunt-ended molecules was shown to noticeably increase in the presence of high concentrations of these macromolecules [36]. These authors pointed out that the effects of macromolecular crowding on blunt-end ligation were correlated with the excluded volume-induced increase in the effective concentrations of the DNA termini for macromolecular joining [36]. In agreement with the notion that high polymer concentrations increase the effective concentration of the ends of the DNA molecules to be joined, macromolecular crowding was shown to increase the rate of nonenzymatic cohesion of the complementary ends of DNA and RNA [37]. In subsequent studies, the Zimmeran group showed that macromolecular crowding (induced by high concentrations of PEG 8000, PEG 20,000, Ficoll 70 or BSA) can efficiently modulate several reactions catalyzed by T4 RNA ligase, such as cyclization of small oligoriboadenylates, the novel formation of large linear products from the oligoriboadenylates, the formation of hybrid molecules by the end-to-end ligation of p(dT)_10_ to single-stranded oligoribonucleotides, and the activation of p(dT)_n_ caused by transfer of an adenylyl moiety from ATP to the oligonucleotides [38].

In addition to the increase in the effective concentration of DNA ends, macromolecular crowding was shown to increase the efficiency of protein binding to DNA [39]. This conclusion was based on the fact that the enzymatic activity of DNA polymerase I of *Escherichia coli* was dramatically increased by high concentrations of crowding agents under otherwise inhibitory conditions resulting from relatively high ionic strength [39]. Furthermore, macromolecular crowding was shown to extend the range of conditions under which DNA polymerase was functional due to the increased binding between the polymerase and its DNA template-primer [40]. In fact, in these experiments, the presence of high concentrations of inert crowding agents promoted nick translation under otherwise strongly inhibitory conditions, such as a range of pH values or temperatures or inhibitory concentrations of urea, formamide or ethidium bromide [40].

In line with these observations, Lavery and Kowalczykowski showed that the addition of high concentrations of PEG or polyvinyl alcohol (PVA) defined the ability of the recA protein to promote homologous pairing and exchange of DNA strands in otherwise non-permissive conditions [41]. These authors also showed that in the crowded environment, the steady-state affinity of recA protein for single-stranded DNA (ssDNA) and the rate of recA protein association with ssDNA-containing secondary structures were noticeably increased [41].

Analysis of the effect of macromolecular crowding on reversed proteolysis revealed that crowded media are able to facilitate proteosynthesis of a polypeptide product with an interacting folding motif such as coiled coil [42]. In the same work, the protease-mediated conversion of some non-covalent protein complexes obtained by limited proteolysis to the native covalent form was shown to be promoted by crowded environment (e.g., the re-formation of native triosephosphate isomerase (TIM) from multiple fragments catalyzed by subtilisin (EC 3.4.21.62)) [42]. However, not all nicked proteins could be ligated under similar conditions, suggesting that this approach might work only if the formation of the native protein results in large compaction [42].

Calcium binding-controlled guanine nucleotide exchange activity of Ras (a member of a class of small GTPases) was dramatically enhanced by addition of high concentrations of either of two macromolecular crowding agents, Ficoll 70 or dextran 70, and this modulated extent of the nucleotide exchange was shown to increase significantly with the concentrations of crowding agents [43].

Catalytic parameters of the monomeric multicopper oxidase, *Saccharomyces cerevisiae* Fet3p (which consists of three cupredoxin-like β-barrel domains and four copper ions located in three distinct metal sites, with site T1 being located in domain 3, and with sites T2 and the binuclear site T3 being positioned at the interface between domains 1 and 3), were remarkably modulated by macromolecular crowding *in vitro* [44]. In this study, different concentrations of macromolecular crowding agent were shown to have rather different effects, with low concentrations being able to increase both *K*_M_ (weaker substrate binding) and *k*_cat_ (improved catalytic efficiency), and with higher concentrations of crowding agent leading to decreases in both catalytic parameters [44]. Although the structural content of Fet3p was not affected by the presence of crowding agents, the thermal stability of this protein was enhanced in the crowded environment. These findings were interpreted in terms of crowding-modulated ordering of a non-optimal substrate-binding site and restricted internal dynamics of the protein [44].

The analysis of the effects of different concentrations of dextrans of various molecular weights on the reaction rates of the hydrolysis of *N*-succinyl-l-phenyl-Ala-*p*-nitroanilide catalyzed by α-chymotrypsin (EC 3.4.21.1) revealed that the volume occupied by the crowding agent, but not its size, plays an important role in controlling the rate of this reaction [45]. The authors also showed that for a dextran with a given molecular weight, a decrease in the crowding agent concentration was accompanied by a *v*_max_ decay and a *K*_m_ increase [45]. Here, the increase in *K*_m_ was attributed to the slowed protein diffusion in the crowded media, and the decrease in *v*_max_ was ascribed to the crowding-enhanced effects of mixed inhibition by product [45]. Enzyme catalytic efficiency of *Plasmodium falciparum* purine nucleoside phosphorylase (EC 2.4.2.1) was inversely affected by the addition of PEGs and dextran, being characterized by the decrease in *k*_cat_ value in the presence of PEGs and dextran [46]. Curiously, decreased substrate binding was observed at low concentrations of crowding agents whereas higher concentrations of PEGs and dextran favored substrate binding [46].

Macromolecular crowding by high concentrations of Ficoll, dextran and PEG 6000 was found to significantly enhance the intrinsic catalytic efficiency of the enterobactin-specific isochorismate synthase (EC 5.4.4.2) by inducing structural change in this enzyme [47]. The analysis of the effect of crowding with PEG on structure and function of the human translation initiation factors, a two-domain DEAD-Box helicase eIF4A, the HEAT-1 domain of eIF4G, and their complex, showed that macromolecular crowding affects activity of these factors involved in human translation initiation [48]. This study showed that a crowding-driven shift of the conformational equilibrium of eIF4A from an inactive open state towards the closed active conformation enhances the ATPase activity of this translation initiation factor. On the other hand, although the folded parts of isolated HEAT1 domain of eIF4G were minimally affected by PEG, the crowded environment was able to significantly alter mobile regions of these proteins [48].

Mitogen-activated protein kinase kinase MEK (MEK MAPKK)-driven phosphorylation of the Tyr and Thr residues within the activation loop of ERK MAPK (mitogen-activated protein kinase ERK) was shown to be dramatically affected by macromolecular crowding with PEG 6000 [49]. In dilute solutions, MEK binds to and phosphorylates ERK to generate tyrosine monophosphorylated ERK (pY-ERK), and then dissociates from pY-ERK, while the crowding environment generates appropriate conditions for the quasi-processive mechanism of ERK phosphorylation by MEK, where after the completion of the first phosphorylation step that generates pY-ERK and due to the restricted diffusion of MEK and pY-ERK, MEK rebinds and processively phosphorylates pY-ERK, producing the tyrosine and threonine bisphosphorylated ERK (pTpY-ERK) [49]. This crowding-enabled biphosphorylation represents a mechanistic foundation of the “distributive phosphorylation model” describing the ability of the ERK MAP kinase pathway to respond to increasing levels of progesterone in an all-or-none or “switch-like” manner, where individual cells in the population exhibit either “on” or “off” status [50].

Although examples considered above suggest that crowding represents a mechanism for modulating the catalytic activities of enzymes, it is important to emphasize that the enzymatic activities of some proteins are not affected by macromolecular crowding. The concentration of added crowding agent (dextran 40) up to 395 mg/mL did not affect binding of a non-natural substrate ester, *p*-nitrophenyl acetate to trypsin (EC 3.4.21.4) [51]. Recently, Vöpel and Makhatadze reported that high concentrations of Ficoll 70 did not perturb the Michaelis constant, *K*_m_, and the catalytic turnover number, *k*_cat_, of three glycolytic enzymes, such as phosphoglycerate kinase (EC 2.7.2.3), glyceraldehyde 3-phosphate dehydrogenase (EC 1.2.1.12), and acylphosphatase (EC 3.6.1.7) [52]. Similar effects of crowding agents on the kinetic parameter, *K*_m_, (where the *K*_m_ changes represented as the ratio of the *K*_m_ in the absence of the crowding agent divided by the *K*_m_ in the presence of the crowding agent ranges from 1.3 to 1.9) were earlier reported for several other monomeric enzymes (e.g., isochorismatase EntB, EC 3.3.2.1, *K*_m_ change of 1.6 [47] and hyaluronate lyase, EC 4.2.2.1, *K*_m_ change of 1.4 [53]).

#### 2.2.2. Ordered Oligomeric Proteins

The mechanisms of the function-modulating effects of macromolecular crowding on oligomeric proteins are different from those affecting monomeric enzymes. In fact, excluded volume is expected to affect an enzymatic activity of a monomeric protein if catalysis is accompanied by changes in the size, or shape of a target enzyme. For the oligomeric protein, the mechanism of the macromolecular crowding action is different. Here, the formation of dimers (or high order oligomers and aggregates) will be preferable in the crowded milieu due to the fact that the excluded volume around each dimer of the protein of interest is smaller than twice the excluded volume of each monomer [15]. In other words, in response to the addition of the bystander molecules, the system will change to minimize the overall crowding by enhancing the association of molecules of interest, thereby reducing the excluded volume [15]. Since the functionality of many proteins depends on their oligomeric state, enhanced oligomerization in crowded environments will obviously affect function of a protein of interest.

One of the first studies on the effect of macromolecular crowding on enzymatic activity was the work of Minton and Wilf [54], where the influence of high concentrations (300 mg/mL) of ribonuclease A (RNase A), β-lactoglobulin, BSA, and PEG 20,000 (poly(ethylene glycol) with the molecular mass of 20,000 Da) on the specific activity of rabbit muscle glyceraldehyde-3-phosphate dehydrogenase (GAPD) was investigated. In the absence of crowding agents, GADP was shown to exist as a mixture of very active monomers and much less active tetramers. Although the activities of monomer and tetramer were not affected by the addition of high concentrations of crowders, the crowded environment shifted the monomer–tetramer equilibrium dramatically favoring the tetramer formation [54]. In a similar way, crowding with high concentrations of PEG 6000 or PEG 35,000 stabilized the bacterial phosphoenolpyruvate:carbohydrate phosphotransferase system (PTS) which catalyzes the uptake and concomitant phosphorylation of glucose (and also regulates catabolite repression), thereby affecting the catalytic activity of this multienzyme complex and its flux-response efficiency [55,56].

Glucose-6-phosphate dehydrogenase (G6P DH) is a dimeric enzyme (EC 1.1.1.49) that catalyzes the oxidation of glucose-6-phosphate (G6P) to gluconolactone-6-phosphate, with NADP+ simultaneously accepting hydrogen to form NADPH. Although the dimer is the active form of G6P DH, the monomeric and tetrameric forms of this protein are inactive. BSA and PEG 6000 were shown to mediate a significant increase in catalytic activity of this protein (caused by crowding-promoted stabilization of active dimer) under conditions promoting its dissociation and inactivation in dilute solutions (incubation at 45 °C for 1 h) [57].

Crowding agent-promoted structural changes were attributed to the enhanced catalytic activity of the endoplasmic reticulum glucosidase II (G-II, EC 3.2.1.84, a multisubunit enzyme that is composed of a catalytic subunit, conserved from yeast to mammals, and a tightly bound noncatalytic His–Asp–Glu–Leu (HDEL)-containing subunit) [58]. Also, both *k*_cat_ and *K*_M_ of hexokinase (a homodimeric enzyme, EC 2.7.1.1) were noticeably decreased (~30%) in the presence of high BSA concentrations due to the conformational change during catalysis and/or diffusion of product from enzyme–substrate complex [59]. Similarly, both Michaelis-Menten constants of NADH oxidation by pyruvate catalyzed by l-lactate dehydrogenase (LDH), *k*_cat_ and *K*_M_, were reduced by high concentrations and large sizes of dextran, suggesting the existence of mixed activation-diffusion control in an enzymatic reaction due to the effect of crowding [60].

On the contrary, Ficolls and dextrans of different sizes (in a variety of sizes ranging from 15 to 500 kDa, in a concentration range 0%–30% *w*/*w*) were shown to reduce the rate of the hydrolysis of *p*-nitrophenyl phosphate by alkaline phosphatase (which is a homodimeric enzyme from bovine intestinal mucosa, EC 3.1.3.1) in a crowder molecular mass-dependent manner, with crowding agents with greater molecular weight possessing high rate reducing capacities [61].

An important role of macromolecular crowding in supporting functional activities in the nucleus was pointed out [62]. In fact, the nucleus is packed with high-molecular chromatin, various ribonucleoprotein particles, associated proteins and specific macromolecular machines that participate in various nuclear processes, such as gene transcription and hnRNA splicing. Therefore, crowding generates a plausible driving force for the assembly and stabilization of these macromolecular complexes [62].

Crowding plays an intricate role in the regulation of the folding-promoting activity of GroEL–GroES chaperone machinery [63]. Here, folding of a target protein occurs in a sequestrated environment of the central GroEL cavity. An intriguing point here is the fact that the displacement of unfolded polypeptide into the “folding” chamber of GroEL requires GroES binding, whereas the resulting native protein leaves GroEL upon GroES release. When folding is slow, each cycle of GroES binding to and dissociation from GroEL is accompanied by the release of non-native polypeptide into the bulk solution. However, this loss of substrate from GroEL was shown to be prevented when the folding reaction was carried out in the presence of macromolecular crowding agents, such as Ficoll and dextran [63].

To understand the molecular mechanisms defining the collaboration between the DnaK system and ClpB, the association equilibrium, biochemical properties, stability, and chaperone activity of the disaggregase ClpB in the absence and presence of an inert macromolecular crowding agent have been recently analyzed [64]. This analysis revealed that macromolecular crowding has a multidirectional effect on the functional activity of this important hexameric chaperone. In fact, the crowded environment shifted the conformational equilibrium of the protein monomer toward a more compact state, dramatically enhanced the association constant of the functional hexamer, stimulated the ATPase activity of ClpB and promoted interaction of this chaperone with substrate proteins and with aggregate-bound DnaK [64].

Crowded environments were shown to modulate the catalytic activity of one of the nonribosomal peptide synthetases (NRPSs), the *Escherichia coli* enterobactin synthetase [65,66]. This protein is a two-module NRPS that includes 2,3-dihydroxybenzoate (DHB)–AMP ligase (EntE), aryl-carrier protein (ArCP) (EntB *C*-terminal domain), and a four-domain protein (EntF) [67]. The premature release of linear precursors/side products, which are abundantly present in the reaction mixture produced by an *in vitro* reconstitution of this complex machine, is suppressed to a negligible level in the crowded environment containing high concentrations of the inert polymers such as Ficoll 70, dextran, PEG, and BSA [65]. The subsequent study showed that this peculiar suppression of the liner side product production is suppressed not due to the stabilization of the quaternary structure of this proteinaceous machine by the crowded environment, but due to the structural changes induced in the *E. coli* NRPS by macromolecular crowding agents [66]. In fact, the authors showed that macromolecular crowding was able to dramatically affect the secondary structure of the major component of the enterobactin NRPS, which is directly associated with the formation of all the side products in the enterobactin synthesis, EntF [66], since the circular dichroism (CD) signal intensity at 210 nm increased significantly with the increase in the crowdedness of the solution [66]. Comparable Ficoll 70-induced structural changes were also detected in the other two components of the enterobactin NRPS, EntB, and EntE [66]. Importantly, the fraction of the side products in the total turnover products generated by the *E. coli* NRPS was shown to decrease exponentially with the increase in the amplitude of structural changes induced in EntF by Ficoll 70, suggesting that the EntF structural changes provoked by the macromolecular crowding agents suppress the production of the side products likely due to the reduced accessibility of the solvent molecules to the active sites of this protein [66].

The catalytic activity of the 3C-like peptidase of the severe acute respiratory syndrome virus (SARS-CoV), a SARS-CoV 3CLpro enzyme which is crucial for viral replication, was significantly enhanced in the presence of macromolecular crowding agents, BSA and PEG [68]. Since in dilute solutions this protein exists as a monomer-dimer mixture, and since only the dimer is proteolytically active, the enhanced catalytic activity of SARS-CoV 3CLpro in a crowded environment was attributed to the crowding-induced stabilization of an active dimeric form [68].

PixD from thermophilic cyanobacterium *Thermosynechococcus elongatus* BP-1 (TePixD, Tll0078) is a 17 kDa protein that consists of the BLUF (sensors of blue light using flavin adenine dinucleotide (FAD)) domain and short helices [69,70]. TePixD is assembled two pentamer rings that form a decamer, and possesses typical photochemistry of the BLUF proteins [71]. The pentamer-decamer equilibrium, which is crucial for the photoactivity of this protein, was shifted toward the more active decamers in the presence of high concentrations of Ficoll 70 [72].

#### 2.2.3. Effect of Macromolecular Crowding on Small-Molecule Substrates

The majority of research on the effect of macromolecular crowding on enzymatic activity is focused on the protein part, with crowding effects on enzymatic reactions being often understood in terms of the crowding-induced changes in enzyme conformation [73]. However, small-molecule substrates can also be affected by macromolecular crowding, especially if these substrates are chemically different and therefore can differently interact with crowding agents [73]. The validity of these considerations was recently confirmed by analyzing the reaction of horseradish peroxidase (HRP) with two small-molecule substrates that differ in their hydrophobicity in crowded media [73]. Aumiller *et al.* showed that the HRP activity in similarly crowded conditions was different when 3,3',5,5'-tetramethylbenzidine (TMB, which is hydrophobic) was used as a substrate as compared to *o*-phenylenediamine (OPD, which is more hydrophilic) [73]. In fact, the reaction rates of HRP with TMB were much more sensitive to the presence of crowding agents than OPD, suggesting that these small-molecule substrates are able differently interact with PEG and dextran, which were used as crowding agents [73]. The ability of small molecular substrates to be involved in weak attractive interactions with crowders is dependent on the chemical nature of both substrates and crowding agents. Such weak attractive interactions, when present, can decrease substrate chemical activity and consequently reduce the enzymatic activity of a protein of interest [73]. Obviously, these observations clearly show that careful consideration of substrate chemistry is needed when enzymatic reactions are analyzed in complex media such as biological fluids [73].

### 2.3. Structural Perturbations of Intrinsically Disordered Proteins in Crowded Milieu

Evidence is rapidly accumulating that many protein regions and even entire proteins lack stable tertiary and/or secondary structure in solution, existing instead as dynamic ensembles of interconverting structures. These naturally flexible proteins and regions are known as intrinsically disordered proteins (IDPs) and intrinsically disordered protein regions (IDPRs). These intricate members of the protein kingdom are abundantly involved in numerous biological processes [74,75,76,77,78,79,80,81,82,83,84,85,86,87,88,89,90,91,92,93,94,95,96,97,98,99,100,101,102,103,104,105,106,107], where they are found to play different roles in regulation of the function of their binding partners and in promotion of the assembly of supra-molecular complexes. To some extent, conformational behavior and structural features of IDPs and IDPRs resemble those of non-native states of globular proteins and might contain collapsed-disorder (*i.e.*, where intrinsic disorder is present in a molten globular form) and extended-disorder (*i.e.*, regions where intrinsic disorder is present in a form of random coil or pre-molten globule) under physiological conditions *in vitro* [76,84,86]. IDPs and hybrid proteins possessing ordered domains and functional IDPRs are very common in any given proteome [76,105,108,109,110]. The functional repertoire of these proteins and functional advantages of being disordered are well-known [74,75,76,78,81,82,86,87,88,92,97,98,100,111,112,113,114,115,116,117,118], and IDPs and hybrid proteins are included to the redefined protein structure-function paradigm [74,76,77,79,80,82,83,84,87,92,93,100]. Finally, IDPs are commonly associated with various human maladies [119].

Since despite the fact that IDPs have large hydrodynamic dimensions in their unbound forms, they contain noticeable residual structure and therefore possess intrinsic propensity for at least partial function-related folding (e.g., as a result of interaction with specific binding partners), it was expected that these proteins would be extremely sensitive to the presence of crowding agents and that macromolecular crowding would promote their effective folding and compaction. This is because of the assumption that the presence of a space-filling substance might significantly affect the protein folding process by favoring a more compact (folded) state over the more extended (unfolded) form.

The effect of high concentrations of crowders on structural properties of several IDPs was analyzed. For example, Flaugh and Lumb [120] established that molecular crowding modeled by the high concentrations (of up to 250 g/L) of the dextrans of average molecular weights 9.5, 37.5, and 77 kDa and Ficoll 70 did not induce significant folding in two IDPs, FosAD and p27ID. FosAD corresponds to the *C*-terminal activation domain of human c-Fos (residues 216–310) and is functional for interacting with transcription factors in whole-cell extract [121]. p27ID corresponds to the cyclin-dependent kinase inhibition domain of the cell-cycle inhibitor human p27^Kip1^ (residues 22–97) and is active as a cyclin A-Cdk2 inhibitor [122]. Both protein domains are intrinsically disordered as judged by circular dichroism (CD) spectra characteristic of the unfolded polypeptide chains, lack of ^1^H chemical-shift dispersion and negative ^1^H-^15^N nuclear Overhauser effects [121,122]. In the presence of macromolecular crowding agents, none of these IDPs underwent any significant conformational change reflected in noticeable changes in either circular dichroism or fluorescence spectra. Therefore, molecular crowding effects are not necessarily sufficient to induce ordered structure in IDPs [120]. Similarly, α-synuclein was shown to preserve its mostly unfolded conformation in the presence of several crowding agents [123] and even in the periplasm of the bacterial cells [124]. Also, analysis of macromolecular crowding on three dehydrins from *Arabidopsis thaliana*, Cor47, Lti29, and Lti30 revealed that, on the contrary to the hypothesis these drought-induced IDPs might acquire a biologically active structure upon dehydration, dehydrins were highly resistant to crowding-induced structural changes, being remarkably stable in their disordered state and being only modestly affected by the solvent alterations [125].

The analysis of FlgM, which is a 97-residue IDP from *Salmonella typhimurium* that regulates flagellar synthesis by binding the transcription factor σ^28^, revealed that approximately half of this IDP gained structure in the crowded environment [126]. Importantly, although free FlgM was mostly unstructured in the dilute solutions, its *C*-terminal half formed a transient helix in the unbound form [127], became structured on binding to σ^28^ [128] and was shown to be folded in the crowded environment [126]. Although the *C*-terminal domain of the histone H1 has little structure in aqueous solution, it became extensively folded in the presence of high concentrations of crowding agents, such as Ficoll 70 and PEG 6000 [129].

Analysis of the conformational behavior of a destabilized mutant of the immunoglobin G binding domain of protein L from the mesophile *Streptococcus magnus* that is mostly unfolded in water but can be folded upon addition of salt, revealed that in the presence of 200 mg/mL dextran 20 this IDP can efficiently fold to a structure that matches the structure of the wild type protein [130]. The authors also showed that the thermodynamic effects of a crowding agent on the folding equilibrium constant for unfolded protein L (ΔΔ*G*_U_ ≈ 2 kJ·mol^−1^) were similar to the crowding effects found on the folded wild type protein and the mutant when prefolded by salt, suggesting that the effect of macromolecular crowding is independent of starting protein stability. Importantly, this study also clearly indicated that crowding can shift the conformational equilibrium toward the folded form when the polypeptide is in the transition region between folded and unfolded states [130].

Small-angle neutron scattering (SANS) with D_2_O-based contrast matching was used to examine the effects of high concentrations of a small globular protein, bovine pancreatic trypsin inhibitor (BPTI), on hydrodynamic dimensions of an intrinsically disordered N protein of bacteriophage λ [131]. In these experiments, to obtain only the scattering signal from the IDP, the λ N protein was labeled with deuterium, whereas the scattering contrast between the solvent and unlabeled BPTI was eliminated by adjusting the D_2_O concentration of the solvent. Although the increase in the BPTI concentration to 65 mg/mL resulted in the detectable decrease in the dimensions of an IDP, further increase in the BPTI concentration to 130 mg/mL did not lead to subsequent decrease in the IDP dimensions [131]. A similar SANS-based study on the effect of high concentrations of BPTI and equine metmyoglobin on the conformational ensemble of a uniformly labeled IDP, the λ N protein in samples with adjusted D_2_O concentration to mask contribution of unlabeled crowding proteins to the scattering profiles revealed that the radius of gyration of this IDP (which for the protein in dilute solution was estimated to be 30 Å) was remarkably insensitive to the presence of crowders, decreasing by a mere <2 Å in the presence of the highest crowder concentrations [132].

Since IDPs do not possess unique structures and comprise highly heterogeneous and dynamic ensembles of conformations, single-molecule spectroscopy, including single-molecule fluorescence detection in combination with Förster resonance energy transfer (FRET), is a highly suitable method for analyzing structural properties of IDPs and investigating the effects of crowded environment on the conformational distributions within their dynamic structural ensembles by looking at one protein molecule at a time. It was also pointed out that the advantage of the single molecule FRET is in its ability to study heterogeneous structural ensemble of suitably labeled IDPs even in the presence of very large concentrations of unlabeled solutes [133]. This technique, where target proteins were labeled with Alexa Fluor 488 and Alexa Fluor 594 as donor and acceptor fluorophores via cysteine residues introduced at suitable positions, was recently applied to study the effect of increasing concentrations of PEG 6000 on the conformational ensembles of four different IDP sequences, such as *N*- and *C*-terminal segments of human prothymosin-α (ProTα-N and ProTα-C), the binding domain of the activator for thyroid hormones and retinoid receptors (ACTR), and the *N*-terminal domain of HIV-1 integrase (IN) [133]. Among a variety of interesting data reported by these authors, one fact was directly related to the topic of this review, namely with increasing concentration of PEG 6000, three of the four disordered sequences (ProTα-C, ProTα-N, and ACTR) were shown to possess a clear overall tendency to collapse in the presence of crowding agents. For the case of IN, which has the least charged and most hydrophobic sequence, only very small compaction was found in the crowded milieu, providing further support to the notion that molecular crowding does not affect all IDPs equally [133].

Calcium binding protein RC_L_ offers the rare opportunity to study the same polypeptide chain under two drastically distinct folding states: as an extended IDP in its apo-form (*R*_H_ of 3.2 nm) and as a compact folded structure in the Ca^2+^-bound form (holo-form, *R*_H_ of 2.2 nm) [134]. RC_L_ is derived from the RTX-containing domain (Repeat in ToXin) of the adenylate cyclase toxin (CyaA) from *Bordetella pertussis*. It was recently shown that although the structural contents of the apo-state and holo-state of RC_L_ were not affected by the crowding agent Ficoll 70, the protein affinity for calcium and thermal stability of both forms were strongly increased by this crowding agent [135].

Cino *et al.* analyzed the effect of macromolecular crowding on structural properties and conformational dynamics of several IDPs with different extents of residual structures by measuring their NMR spin relaxation parameters in the absence and presence of 160 mg/mL of Ficoll 70 [136]. ^1^H–^15^N HSQC spectra of uniformly ^15^N labeled prothymosin α (ProTα; human isoform 2), thyroid cancer 1 protein (TC-1; human), and α-synuclein (human isoform 1) in dilute and crowded solutions suggested that all three proteins remain mostly disordered under crowded conditions and retained their segmental motions on the nanosecond timescale [136]. These authors also showed that the crowded environment exhibited differential effects on the conformational propensity of distinct regions of an IDP [136].

Based on the examples considered in this section, one may conclude that IDPs could be grouped into two classes, foldable and non-foldable, based on their response to the crowded environment. Foldable IDPs can gain structure in crowded environment (and, thus, inside the living cells) likely due to the crowding-induced formation of a hydrophobic core. Non-foldable IDPs remain mostly unstructured at the crowded conditions. Some of these non-foldable by crowding IDPs may require another protein (or DNA, or RNA, or some other natural binding partners) to provide a framework for structure formation. FlgM clearly represents a unique case of the two-faced Janus, where the first face exemplified by the *C*-terminal half of FlgM is structured in the crowded environment, and the second face exemplified by the *N*-terminal half of FlgM does not become structured at physiologically relevant solute concentrations [126].

### 2.4. Effects of Macromolecular Crowding on Shape of Protein Molecules

Obviously, high concentrations of inert bystander molecules might affect any chemical equilibrium involving changes in the accessible volume. Therefore, among the potential targets that might be influenced by the presence of crowding agents are multidomain proteins existing in close and open functional states, or proteins undergoing transitions between elongated and more compact forms.

Combining a set of *in vitro* and *in silico* methods, Homouz *et al.* analyzed the effect of macromolecular crowding on structural changes and shape of an aspherical protein VlsE from *Borrelia burgdorferi* [12]. VlsE possesses an elongated football shape with a helical core surrounded by floppy loops at each end [137] and unfolds in a two-state manner both kinetically and in equilibrium [138,139]. The authors showed that the folding kinetics of this football-shaped protein is accelerated in the presence of high concentrations of Ficoll 70 [12]. Also, there was a noticeable shift in the transition midpoint to higher urea concentrations, and the unfolding-free energy increased in magnitude in the presence of Ficoll 70 [12]. Curiously, increasing concentrations of Ficoll 70 resulted in a significant increase in the helical secondary structure of this protein (from 50% in the absence of crowding agent to 80% in the presence of 400 mg/mL of Ficoll 70). Addition of high Ficoll 70 concentrations to the solution of VslE in the presence of urea resulted in the formation of noticeable non-native β-structure [12]. *In silico* modeling revealed that crowding induces the transition from the football-like shape to a more compact bean-like shape combined with the noticeable increase in helical structure. A temperature increase resulted in the appearance of a spherical species which was formed by “breaking” the bean-like structure in the middle leading to the most compact state with lost native helical interactions and gained nonnative interactions corresponding to β-strand contacts [12]. These crowding-induced shape changes of VslE were suggested to be of biological significance since they lead to exposure of a hidden antigenic region [12].

Application of an analogous approach based on the combination of *in silico* (coarse-grained molecular simulations) and experimental (fluorescence resonance energy transfer techniques) methods revealed that calmodulin (CaM), a dumbbell-shaped molecule that contains four EF hands (two in the *N*-lobe and two in the *C*-lobe), is converted into more compact structures in the presence of crowding agents [140]. These analyses also showed that crowding was able to stabilize several different compact conformations of CaM, suggesting that structure of this proteins is characterized by the inherent plasticity [140]. In a subsequent study, the same authors used combined methods of computer simulation and experiments to show that the conformation, helicity and the EF hand orientation of CaM are affected by the increased levels of macromolecular crowding agents, suggesting that crowding might contribute to the promiscuous behavior of calmodulin in target selection inside cells [141].

When a similar combined approach was applied to phosphoglycerate kinase (PGK) the existence of noticeable compaction of this protein accompanied by the dramatic increase in its enzymatic activity in the presence of Ficoll 70 was noted [142]. The coarse-grained molecular simulation analysis suggested that PGK can exist in three compact ensembles: crystal structure ensemble (that corresponds to open extended form of a protein), collapsed crystal structure ensemble (that represents a compact closed form), and spherical compact ensemble, where the ADP and diphosphoglycerate binding sites of PGK come together. Since the proximity of these two active sites is crucial for ATP synthesis, macromolecular crowding defines an absolutely novel, hinge motion-independent mechanism by which this enzyme operates in crowded *in vivo* conditions [142].

### 2.5. Conformational Behavior and Folding of Proteins in Crowded Environment

Besides direct effects on protein structure and function, macromolecular crowing can affect the protein folding process, influence its conformational equilibrium and thereby modulate the conformational behavior and stability of a protein. In fact, the application of the excluded volume model to a protein folding process (*i.e.*, a transition from a less compact unfolded form to a more compact folded state) suggests that the presence of a crowding agents might significantly affect the protein folding process by favoring a more compact (folded) state over the more extended (unfolded) form, and these folding–promoting effects are expected to increase exponentially with the increase in the concentration of the crowding agent [15]. In agreement with these predictions, numerous studies clearly showed that macromolecular crowing can affect the protein folding process, influence protein conformational equilibrium and thereby modulate the conformational behavior and stability of a protein.

#### 2.5.1. Effects of Crowded Milieu on Protein Conformational Stability

When a single-molecule method based on atomic force microscopy (AFM) was used to investigate the effects of macromolecular crowding on the forces required to unfold individual protein molecules, it was concluded that the mechanical stability of a protein can be dramatically increased by macromolecular crowding [143]. For example, the average mechanical force required to unfold a single ubiquitin molecule was shown to increase from 210 pN in the absence of dextran to 234 pN in the presence of 300 mg/mL dextran [143].

Although the secondary structure of a small single-domain protein cytochrome *c* was not affected by the addition of Ficoll or dextran (0–400 mg/mL, pH 7), thermal stability of this protein was noticeably increased (5–10 °C) in the presence of Ficoll 70 or dextran 70. This effect was further enhanced when low concentrations of guanidine hydrochloride (GdmHCl) that destabilize the protein were present, and the stabilizing effects of a smaller dextran, dextran 40, were significantly greater (10–20 °C) [144]. Based on these and other studies the authors suggested “that protein size, stability, conformational malleability, and folding routes, as well as crowder size and shape, are key factors that modulate the net effect of macromolecular crowding on proteins” [144]. In agreement with this statement, recent analysis of the effect of dextran 20 on conformational stability and structural features of hyperthermophilic (S16Thermo) and mesophilic (S16Meso) homologues of the ribosomal protein S16 revealed that crowding induces structural changes in a protein-dependent manner [145].

Thermal stability of folded apoflavodoxin was shown to increase by about 3 °C in the presence of high concentrations of dextran 20. The addition of this crowding agent to the solution of GdmHCl-unfolded apoflavodoxin resulted in a decrease in protein volume of about 29%. Refolding efficiency of this protein in crowded environments *in vitro* was dramatically hampered due to the severe aggregation of the compact off-pathway folding intermediate [146].

Analysis of heat-induced denaturation of the three small well-folded globular proteins, RNase A from bovine pancreas, lysozyme from chicken egg white, and holo α-lactalbumin (α-LA) from bovine milk, in the presence of different Ficoll 70 concentrations (0, 100, 200, 300 and 400 g/L) at different pH values (7.0, 6.0, 5.0 and 4.0) revealed that the *T*_m_ (midpoint of denaturation) of all the proteins remained unperturbed in the presence of Ficoll 70 at physiological pH [147]. At moderately acidic pH (pH 4.0), Ficoll 70 had a stabilizing influence on RNase A and α-LA, whereas the measured thermodynamic parameters of lysozyme at low pH values remained practically unchanged upon addition of Ficoll 70 relative to that in dilute aqueous solutions [147]. Also, in agreement with these thermodynamic data, structural characterization of the heat denatured state by far-UV CD revealed that the addition of Ficoll 70 to RNase A and α-LA at pH 7.0 and 4.0 resulted in an increase in the residual structure, and no such increase in the residual structure was detected for the temperature-unfolded state of lysozyme [147]. Therefore, the thermodynamic and structural analyses of these three proteins confirmed that the effects of macromolecular crowding are dependent on the peculiarities of a given protein-crowder system [147].

The structural analyses using far-UV CD and intrinsic fluorescence spectroscopy revealed that the thermal stability of creatine kinase (CK) was noticeably enhanced in the presence of Ficoll 70 [148]. Here, the increase in the concentration of this crowding agent resulted in an increase in the transition temperature *T*_m_ with constant enthalpy change Δ*H*_u_ of the thermal denaturation process [148]. The authors also reported that crowding increased the compaction degree of both native and temperature-denatured states of CK, with the denatured ensemble undergoing more noticeable compaction [148].

In agreement with the important notion that activity of oligometic proteins can be noticeably modulated by macromolecular crowding mostly via the crowding-induced stabilization of their oligomeric structures, the detailed analysis revealed that crowding agent Ficoll 70 affected the unfolding behavior of an oligomeric protein, human cpn10 [149]. Similar to its analogue GroES in *E. coli*, human cpn10 is a heptameric protein consisting of seven identical β-barrel subunits assembled into a ring. Using far-UV CD, tyrosine fluorescence, NMR, and cross-linking experiments the authors investigated the influence of Ficoll 70 on thermal and chemical stability of this protein and on its heptamer-monomer dissociation constant. These analyses revealed that the heptameric structure of cpn10 was noticeably stabilized in the presence of high Ficoll 70 concentrations (300 mg/mL), where the heptamer–monomer equilibrium constant was shifted in the direction of the heptamer resulting in significant increase in thermal and thermodynamic stabilization of the heptameric structure, decrease in its dissociation, and the related decrease in the kinetic unfolding rates [149].

In dilute solutions, recombinant human brain-type creatine kinase (rHBCK) is known to be efficiently inactivated by 0.5 M GdmHCl at 25 °C [150]. However, the inactivation process was dramatically slowed down by high concentrations of PEG 2000 or dextran 70. For example, in the presence of 200 mg/mL PEG 2000, ~35.33% of rHBCK activity was retained after 4 h of incubation at 0.5 M GdmHCl and 25 °C, whereas no rHBCK activity was observed when protein was incubated in the absence of macromolecular crowding agents [150].

Zhai and Winter used FTIR spectroscopy to analyze the effect of dextran on the temperature and pressure-dependent phase diagram of ribonuclease A (RNase A) [151]. These authors showed that the crowded dextran solutions markedly prohibited both temperature-induced equilibrium unfolding of RNase A and unfolding of this protein induced by pressure [151]. For example, the pressure-induced unfolding of RNase A in dilute solutions starts at around 3.5 kbar and is completed at ~5.5 kbar (midpoint *p*_m_ = 4.76 kbar), whereas addition of 10, 20 and 30 wt % of dextran increased the unfolding pressure from 4.76 to 5.34, 5.97 and 7.05 kbar, respectively [151]. In another study, FTIR, small-angle X-ray scattering, and calorimetric measurements have been applied to look at the effect of macromolecular crowding by Ficoll on the temperature- and pressure-dependent stability diagram and folding reaction of a model protein Staphylococcal nuclease (SNase) [152]. The authors found that in 30 wt % Ficoll solutions, both temperature- and pressure-induced equilibrium unfolding processes were significantly shifted to higher temperatures and pressures [152]. Also, the refolding rate of SNase was shown to be markedly decreased in the presence of Ficoll [152].

#### 2.5.2. Altered Conformational Behavior of Proteins in Crowded Environments

Actin is the most abundant protein in the eukaryotic cells, where the monomeric actin concentrations are within the 12–300 μM range [153]. Being found in almost every living cell, this globular multifunctional protein is most common in the muscle cells, where its concentration ranges from 230 to 960 μM [153]. Among various functional and structural features of actin are its ability to exists as a monomer known as G-actin (under low ionic strength conditions) or a single-stranded polymer, the so called fibrous form of actin, or F-actin (which results from the polymerization of G-actin in the presence of neutral salts), or an inactive form that lacks the ability to polymerize and can be produced by the release of cation by EDTA or EGTA treatment [154,155,156] among other means. Addition of PEG 6000 was shown to efficiently decrease the rate of the EDTA-induced denaturation of G-actin and increased the temperature at which irreversible conformational changes occur in the actin monomer [157].

Reduced and carboxyamidated RNase T1 (TCAM) is substantially unfolded and is not catalytically active in aqueous media [158]. However, the addition of macromolecular crowding agents (400 mg/mL dextran 70) resulted in the appearance of the catalytically active protein. Since the activity of this crowding-induced form accounted for approximately 16% of the total available TCAM in solution, the authors concluded that macromolecular crowding agents are only modestly effective in promoting folding of this IDP [158].

Noticeable folding of acid-unfolded cytochrome *c* at pH 2.0 was induced by the addition of high concentrations of dextran [159]. Structural characterization of this crowding-induced conformation revealed its close resemblance to the molten globule-like state suggesting that this compact partially folded intermediate is stabilized relative to the fully unfolded form in crowded environments [159]. Similarly, the addition of 35% PEG 20,000 or Ficoll 70 to the solution of the unfolded ribonuclease A (RNase A) in a 2.4 M urea solution at pH 3.0 resulted in the formation of a native compact conformation [29]. The transition of acid-unfolded apomyoglobin to salt-induced molten globular form was promoted by the increasing concentrations of an inert polymer, dextran, which also enhanced stability of this molten globule toward the heat-induced and cold-induced unfolding [160].

#### 2.5.3. Structural Changes Induced in Globular Proteins by Macromolecular Crowding

Secondary structure and conformational stability of the 148-residue single-domain α/β protein, *Desulfovibrio desulfuricans* apoflavodoxin were enhanced by Ficoll 70 in a crowding agent concentration-dependent manner [161]. For example, high concentrations of Ficoll 70 dramatically increased the thermal stability of apoflavodoxin (Δ*T*_m_ of 20 °C at 400 mg/mL; pH 7) [161]. Also, the presence of macromolecular crowding agents was shown to lead to the increased amount of secondary structure in the folded states of two structurally-different proteins, α-helical VlsE and α/β flavodoxin [162]. The authors showed that the structural content of flavodoxin and VlsE was enhanced by 33% and 70%, respectively, in 400 mg/mL Ficoll 70 (pH 7, 20 °C) [162]. This crowding-induced “over-folding” was also accompanied by the noticeable increase in the conformational stability of these proteins [162].

Pielak *et al.* investigated the effect of macromolecular crowding on folding and stability of the globular protein chymotrypsin inhibitor 2 (CI2) in the presence of high concentrations (300 mg/mL) of 40 kDa poly(vinylpyrrolidone) (PVP) [163]. In this study, the structure of the I29A/I37H variant of CI2 in the absence and presence of PVP was probed by hydrogen–deuterium exchange in NMR settings. The authors show that the addition of PVP is associated with some structural rearrangement only in the loop and turn regions of CI2. At the next stage, the stability of CI2 was quantified by the amide-proton exchange. This analysis revealed that the addition of crowding agent resulted in up to a 100-fold increase in the CI2 stability. The residue-by-residue amide-proton exchange measurements suggested that crowding had the largest effects on opening reactions that expose the most surface area but had little or no effect on regions that were solvent-exposed in both the native and open states, supporting the notion that macromolecular crowding stabilizes the native state of a globular protein by destabilizing the unfolded states [163,164].

#### 2.5.4. Protein Folding in Crowded Milieu 

The kinetic study of the unfolding-refolding reaction of a redesigned apocytochrome *b*_562_ revealed that the folding rate of the protein in mild crowded environments (in the presence of 85 mg/mL of PEG 20,000) was significantly accelerated, whereas the unfolded rate remained mostly unchanged [165].

Folding of reduced hen lysozyme was not affected by crowding with Ficoll 70 or dextran 70, whereas the refolding process of the unfolded and reduced protein was almost completely abolished due to the protein aggregation at high concentrations of crowding agents [2]. Similarly, refolding of glucose-6-phosphate dehydrogenase (G6PDH) and protein disulfide isomerase was shown to be hampered by macromolecular crowding due to the formation of soluble aggregates [166]. In other words, it looks like macromolecular crowding enhances protein aggregation, at the expense of correct folding [9].

Curiously, the addition of the protein folding catalyst, protein disulfide isomerase (PDI), to the refolding mixture prevented significant portions of the reduced lysozyme from aggregation [2]. These protective effects were more pronounced in the crowded environment and lesser amounts of PDI were needed to promote efficient protein folding under these conditions [2]. Subsequent analysis of the oxidative refolding of hen lysozyme in the absence and presence of high concentrations of inert crowders (BSA and Ficoll 70) suggested that crowding did not have a significant effect on the energetics of the protein folding reaction [167]. This important conclusion was based on the observation that the refolding in the absence and presence of Ficoll was accompanied by accumulation of very similar folding intermediates [167]. Another important finding of this study was the fact that the fast folding phase was accelerated, whereas the slow phase was substantially delayed in the presence of high concentrations of macromolecules [167]. These findings suggested that there was a preferential stabilization of compact states over more unfolded states [167]. The conclusion was supported by the direct observation of significant increase in protein stability in the presence of high concentrations of crowding agents [159].

It is expected that macromolecular crowding might affect protein folding by influencing the hydrodynamic volume of the unfolded state. In other words, macromolecular crowding might provide an indirect stabilizing effect to the folded states of proteins due to destabilization of the more extended and malleable denatured states [168,169]. The existence of direct effect of macromolecular crowding on the unfolded state of a protein, forcing it to become more compact at crowded conditions, has been demonstrated using the crowding-induced changes in the efficiency of the Förster resonance energy transfer for the urea-unfolded small ribosomal protein S16 labeled with boron-dipyrromethene (BODIPY) [170]. This analysis revealed that in the presence of dextran, the unfolded ensemble of S16 was more compact than in the dilute solutions, and this partially collapsed unfolded form possessed noticeable residual structure, as probed by far-UV CD [170].

The analysis of the global effects of macromolecular crowding on rates of protein unfolding and refolding revealed that although macromolecular crowding agents rarely affect protein unfolding rates, they are able to noticeably accelerate refolding rates of urea-unfolded *Borrelia burgdorferi* VlsE [12], urea-unfolded reduced lysozyme [167], and wild-type chymotrypsin inhibitor 2 (CI2) (*t*_1/2_ = 12 ms) and its faster (*t*_1/2_ = 2 ms) and slower (*t*_1/2_ = 350 ms) folding mutants [171], as well as the rates of the oxidative refolding of reduced and denatured hen egg white lysozyme [172].

The effect of high concentrations of dextran 70 (200 g/L) and Ficoll 70 (200 g/L) on the thermal stability and folding-unfolding kinetics of three small folding motifs (*i.e.*, a 34-residue α-helix, a 34-residue cross-linked helix-turn-helix, and a 16-residue β-hairpin) was analyzed to understand how macromolecular crowding can affect folding capability of short independently foldable structural motifs [173]. Although the helix-coil transition kinetics have been shown to depend on viscosity, folding of helical motifs was not affected by crowding. On the contrary, crowding significantly slowed down the folding rate of the shortest β-hairpin peptide [173].

The importance of macromolecular crowding for protein folding was also demonstrated in a more complex process, the refolding and assembly of tetradecameric GroEL from urea-unfolded protein monomers [174]. Here, an important synergism between nucleotides and crowders was observed. In fact, urea-unfolded GroEL failed to gain native oligomeric structure alone, or in the presence of crowding agents, and was able to only partially refold in the presence of nucleotides (ATP or ADP). However, the simultaneous presence of high concentrations of model crowding agents (Ficoll 70, dextran, or BSA) and ATP in the refolding mixture induces dramatic enhancement in the reactivation of GroEL, as compared with the folding-assembly efficiency with only ATP [174].

A detailed analysis of refolding process of dihydrofolate reductase, enolase, and green fluorescent protein in the absence and presence of crowding agents revealed that although these proteins can fold spontaneously in dilute solutions, they lose this ability in a crowded environment [175]. These proteins were trapped in a form of soluble, protease-sensitive non-native species (presumably in a form of soluble oligomers), but regained the ability to fold in the presence of the complete GroEL/GroES chaperonin system, and this chaperonin-mediated folding was triggered by ATP hydrolysis [175]. These data clearly suggested that folding of many proteins inside the cell might be strongly dependent on assistance by chaperonins [175]. Similarly, the efficiency of the refolding of homotetrameric d-glyceraldehyde-3-phosphate dehydrogenase (GAPD) in the crowded environment was noticeably increased in the presence of GroEL [176].

Hong and Gierasch investigated the effect of Ficoll 70 on the folding landscape and the denatured state of a model β-rich protein, cellular retinoic acid-binding protein I (CRABP I) [177]. Although the equilibrium stability of this protein was mostly unaffected by crowding, the authors established the existence of noticeable crowding-induced changes in compaction degree of its denatured state evidenced by the decreased water-accessible surface leading to the ~15% decrease in the change in unfolding free energy with respect to urea concentration (the *m*-value) and by the reduction of the accessibility of the Trp and Cys residues to iodide quenching and chemical modification, respectively [177].

Analysis of the effect of increasing levels of crowding agents dextran (sizes 20, 40, and 70 kDa) and Ficoll 70 on stability (thermal and chemical perturbations) and folding kinetics (chemical perturbation) of *Pseudomonas aeruginosa* apoazurin revealed that independently of the method of perturbation (chemical or thermal), addition of the increasing amounts of crowding agents resulted in the noticeable stabilization of this protein evidenced by an increase in the midpoint of corresponding unfolding transitions [178]. The cooperativity of these equilibrium transitions was almost unperturbed by crowding agents. Comparison of apoazurin folding dynamics in dilute solution with that in the presence of various amounts of Dextran 20, as a function of GdmHCl concentration at 20 °C revealed that folding and unfolding reactions of this protein retained their two-state nature both in the presence and absence of various concentrations of Dextran 20 [178]. This analysis also showed that with Dextran 20 the folding rate of apoazurin was increased, whereas the unfolding rate constants were mostly unchanged [178]. Comparison of the effects of increasing amounts of Dextran 20 on the apoazurin refolding/unfolding rate constants at six different GdmHCl concentrations revealed that the refolding rate constants increased linearly with crowding whereas there was no effect on apoazurin unfolding rate at any Dextran concentration below 200 mg/mL [178].

The kinetic analysis of the effect of Ficoll 70 on folding-unfolding behavior of PGK showed that the increase in the crowding agent induces non-linear changes in the folding relaxation rate and that the optimum Ficoll 70 concentration exists for fastest folding [142]. The authors concluded that the crowding agent-induced stabilization of the unfolded state relative to the transition state leads to the slowing down of PGK folding below the optimum Ficoll 70 concentration, whereas increase in solvent viscosity defines the slowed down folding of this protein above the optimum concentration of the crowding agent [142].

Addition of 220 mg/mL dextran 70 to the reaction mixture where the reduced cytochrome *c* folds from the GdmHCl-induced unfolded ensemble resulted in a noticeable change of the kinetic profile [179]. In fact, in dilute phosphate buffer, folding of GdmHCl-induced cytochrome *c* involves kinetic partitioning, where one fraction of molecules folds rapidly, on a time scale identical to that of reduction, whereas the remaining population folds more slowly. In crowded medium, the population of the fast folding step was greatly reduced suggesting that macromolecular crowding alters unfolded cytochrome *c* such that access to fast-folding conformations is reduced [179].

A set of 13 mutants of a *Desulfovibrio desulfuricans* apoflavodoxin was used to analyze with residue-specific resolution the effect of crowded conditions *in vitro* on the time-resolved folding reaction of this small parallel α/β protein [180]. In dilute solutions, refolding of apoflavodoxin involves an initial misfolded species that rearranges in a rate-limiting process to the native structure [181]. This initial misfolding is determined by competition between the two sides of the central β-sheet of this protein (*i.e.*, β2β1β3 side *versus* β3β4β5 side of the same β-sheet) [181]. In the presence of 100 mg/mL of Ficoll 70, all analyzed mutants were noticeably stabilized against thermal denaturation [180]. Earlier kinetic analysis of the folding-unfolding behavior of the wild type apoflavodoxin revealed that although unfolding speed was not affected by crowding, the final refolding step became faster in the presence of Ficoll 70 [182]. Mutations did not affect the general folding mechanism, since all mutants showed an initial burst stage (which corresponds to the initial misfolding) followed by a slower single-exponential folding step both in dilute and crowded solutions. However, in the presence of Ficoll 70, the amplitude of this initial burst stage was dramatically reduced for most variants, suggesting that macromolecular crowding reduces topological frustrations in the early stages of apoflavodoxin folding and thereby reduces misfolding of this protein [180]. Curiously, many mutants were shown to possess significantly lower ϕ-values in Ficoll 70 than in buffer, implying a more unfolded-like (or less native-like) folding-transition-state structure, as compared to that in dilute solutions [180]. Therefore, the initial topological frustration that causes protein misfolding is reduced in a crowded environment. As a result, subsequent folding requires less ordering in the transition state, and the β-strand 1 becomes more important in guiding the process [180]. Based on the observed reduced burst phases and lower ϕ-values for the folding-transition state under crowded conditions the authors made an important conclusion on the effect of macromolecular crowding on the folding funnel of this protein, suggesting that in a crowded environment, this folding funnel is narrower at the top (reduced conformational entropy) with smoother walls (*i.e.*, less misfolding and traps) as compared to that in dilute solutions [180].

In-cell NMR was used to evaluate the capability of the cytoplasm of *Escherichia coli* to promote folding of a destabilized globular protein [183]. In this work, a mutant form of protein L (ProtL), a 7 kDa globular protein from the mesophile *Streptococcus magnus* with seven lysine residues replaced by glutamic acids was expressed in *E. coli*. Although the wild-type protein is characterized by relatively high conformational stability as indicated by the fact that just 0.1% of its molecules populate the denatured state in dilute buffer at room temperature, 84% of the ProtL mutant populate the denatured state under the same conditions [183]. This mutation-induced destabilization was attributed to the variant’s decreased hydrophobic surface area, not the increase in negative charge and was shown to be efficiently overcame by adding of Na^+^ or K^+^ salts in dilute solutions [183]. Mutant ProtL remained mostly unfolded in the cytoplasm even when the salt concentration inside *E. coli* was increased [183]. These observations were interpreted in terms of the presence of some nonspecific interactions between the protein and cytoplasmic components. Therefore, stabilizing excluded-volume effects defined by the crowded conditions can be ameliorated by nonspecific interactions between cytoplasmic components [183].

A work of Ye *et al.* represents a standalone study on the effect of macromolecular crowding of refolding and membrane insertion of outer membrane proteins (OMPs) [184]. The authors analyzed the effect of the macromolecular crowding condition on OMP membrane insertion using *Escherichia coli* OmpA and OmpT as model proteins and Ficoll 70 as the crowding agent [184]. The presence of Ficoll 70 significantly slowed down the rates of refolding and membrane insertion of OmpA while had little effect on refolding and membrane insertion of OmpT. Since the specific feature of OmpA is the presence of a large periplasmic domain, the authors created a truncated OmpA version that contained only the transmembrane domain (OmpA171). Although in the absence of crowding agent, OmpA171 and full-length OmpA had similar folding rates, in the presence of 20% Ficoll 70, full-length OmpA refolded much slower than OmpA171 [184]. However, the OmpA171 folding efficiency was significantly decreased in comparison with that of OmpA. These data indicated that the periplasmic domain plays a dual role in refolding-insertion process of OmpA, slowing down the rates, while improving the efficiency, of OmpA folding and membrane insertion under the crowding condition [184].

### 2.6. Protein Assembly in a Crowded Environment

In the case of interacting molecules, the association equilibrium is expected to be affected by the presence of crowding agents [15]. Here, the associating macromolecules of interest and the crowder molecules exclude each other from their neighborhoods. As already mentioned, the excluded volume around each dimer of the macromolecule of interest is smaller than twice the excluded volume of each monomer. Therefore, the formation of dimers (or high order oligomers and aggregates) will be preferable in the crowded milieu, since to keep the overall entropy as high as possible, the system that includes solvent, high concentration of bystander molecules, and some amount of associating molecules of interest will tend to minimize the total excluded volume (or, alternatively, to restore some of the available solution volume lost through the addition of the crowder). In other words, in response to the addition of the bystander molecules, the system will change to minimize the overall crowding by enhancing the association of molecules of interest, thereby reducing the excluded volume [15].

Several facts listed below confirm that various biological equilibria can be shifted by high concentrations of non-specific polymers affecting the various classes of reactions that lead to the assembly of proteins and protein complexes [11].

Macromolecular crowding was shown to efficiently promote assembly of *E. coli* 60S ribosomal particles from the isolated 30S and 50S ribosomal subunits and increase a further interaction between 70S particles and form the 100S dimer [185]. Binding of hepatic aldolase B to the hepatocyte matrix [186], interaction of rat brain hexokinase with mitochondria from rat liver or yeast [187,188], self-assembly of the bacterial cell division protein FtsZ to dimers [189], long rod-like linear aggregates [190] and monomer-thick ribbons [191], formation of secretory granules, which are the storage place for many protein hormones [192], the self-assembly of capsid protein CA of human immunodeficiency virus type 1 (HIV-1) *in vitro* [193], the formation of a nucleoprotein complex between the bacterial virulence protein VirE2 and single stranded DNA (ssDNA) [194], binding of monomeric RepA protein encoded in the Pseudomonas pPS10 replicon to DNA [195], interaction between bovine milk xanthine oxidase and bovine erythrocyte copper, zinc-superoxide dismutase [196], self-association of polynucleosome chains [197], interaction of protein p6 from *Bacillus subtilis* phage phi29 with double-stranded DNA [198], binding of ligands to a receptor near membranes [199], formation of encounter complexes [200], formation of decamers of BPTI [201], homodimerization of apomyoglobin [202], polymerization of deoxyhemoglobin S [203], polymerization of actin [204], enhanced specific interaction between ubiquitin and UIM1 and between cytochrome *c* and cytochrome *c* peroxidase at the expense of nonspecific transient encounter complexes [205], as well as several other assembly-related processes were influenced by macromolecular crowding. However, high concentrations of PEG 600, PEG 1000, PEG 8000, and dextran 6 did not affect the formation of two high-affinity heterocomplexes (TEM1-β-lactamase with β-lactamase inhibitor protein (BLIP) and barnase with barstar) [206]. Also, in the case of the weak binding pair CyPet–YPet (which are the GFP variants) aggregation, and not enhanced dimerization, was detected in concentrated PEG solutions [206].

Formation of the filaments from metabolic enzymes was proposed to serve as a general protective mechanism that allows cell to inactivate and store key metabolic enzymes during a state of advanced cellular starvation [207]. Using filament formation by the enzyme glutamine synthetase (Gln1) as an illustrative example, Petrovska *et al.* showed that the filament assembly is a highly cooperative process, which is strongly dependent on macromolecular crowding and involves back-to-back stacking of cylindrical homo-decamers of Gln1 into filaments that further associate laterally to form higher order fibrils [207].

The assembly of the five-protein core of the DNA replication complex of bacteriophage T4 that includes the DNA polymerase (gp43), the T4-coded single-stranded binding protein (gp32), and three proteins known collectively as the polymerase accessory proteins, gp44, gp62, and gp45, was dramatically enhanced in the presence of high concentrations of inert macromolecular solutes [208]. Although under typical physiological conditions the assembly of the active T4 holoenzyme requires hydrolysis of ATP by the gp44/62 complex, processive T4 holoenzyme-like DNA synthesis can be obtained without hydrolysis of ATP by simply adding extremely high concentrations of gp45 to the T4 DNA polymerase (gp43) [209]. Importantly, the amount of gp45 needed for the formation of active gp43-gp45 heterodimer can be noticeably decreased by the addition of the macromolecular crowding agent PEG [209]. In line with these observations, subsequent study confirmed that the requirement for the gp44/62 complex can be completely eliminated by macromolecular crowding [210]. A logical continuation of these studies was an important notion that macromolecular crowding can be used to identify weak interactions within the DNA replication complex [211].

Besides paying an important role in the action of the DNA replication complex of bacteriophage T4 (see above), the T4 replication accessory protein gp45 belonging to the family of “sliding clamp” proteins also serves as transcriptional activator of phage T4 late genes [212,213,214]. In this activity, the DNA-tracking state of gp45 is determined by the assembly of a ring-form trimer around DNA [215]. Although under the uncrowded conditions this tracking state was rather transient due to the dynamics of spontaneous ring opening, the addition of PEG noticeably stabilized the DNA-tracking state of this protein [215].

It was also shown that the formation of bundles of actin filaments [216], the extent of actin polymerization [157,204], the efficiency of interaction between the gycolytic enzymes and cytoskeletal structures [217,218] and myofibrils [218], conversion of actin bundles into actin filaments [219], and the effects of ancillary cytoskeletal proteins (such as caldesmon, tropomyosin, and filamin) on actin bundle formation were dramatically enhanced in the crowded environment [219]. These observations led to the hypothesis that the organization of the cytoskeleton (e.g., the reversible conversion of actin filaments into actin bundles), and interactions of ancillary proteins with the cytoskeleton, are under macromolecular crowding control [219,220]. Similarly, macromolecular crowding affected the architecture of collagen hydrogels, tuning the rate of collagen nucleation and fiber growth, affecting fiber diameter and organization, and generating gels that are twice as resistant to mechanical stress as the controls [221]. Also, the gel pore size, protein permeability, transparency, and resistance to enzymatic degradation were shown to be tunable by the adjustments of crowding levels during collagen assembly [221].

Macromolecular crowding was proposed to be responsible for the enhanced tendency of soluble macromolecules to associate with membrane proteins that defines the ability of small fractional changes of cell volume to possess large increases in transporter-mediated ion flux across cell membranes [222]. This crowding-controlled regulation of cellular volumes is based on the balance between the rates of phosphatase-catalyzed activation and kinase-catalyzed inactivation of the transporter, where the inhibition of kinase relative to phosphatase activity leading to the increased concentration of the active form of the transporter might be related to cell swelling [222]. The effects of dextrans, carbohydrates, and PEGs of different sizes on the volume of the macrophage nucleus were studied in the permeabilized cell model [223]. This model revealed that nuclei swelled in the presence of small solutes and shrank reversibly in the presence of larger solutes irrespectively of their chemical nature, suggesting that the size and structure of the nucleus can be directly influenced by high concentrations of macromolecules [223].

The assembly-promoting potential of crowding agents is expected to depend on the size of crowding agents and the relative dimensions of interacting proteins [28]. In agreement with this model, macromolecular crowding differently affected the polymerization of the HIV-1 capsid protein CA and inhibition of this process by short peptides, or inhibition of interaction between the foot-and-mouth disease virus (FMDV) and receptor molecules on the host cell membrane by short peptides [224]. In fact, although the assembly of the FMDV) and receptor molecules on the host cell HIV-1 capsid protein CA to capsid-like particles was accelerated in the presence of high concentrations of Ficoll 70 and dextran 10 [193], inhibition of this process by a small *C*-terminal domain (CTD) of protein CA (which is responsible for CA dimerization and, in its isolated form, is able to efficiently inhibit the *in vitro* assembly of the HIV-1 capsid) or by a short CTD-binding dodecapeptide CAI was efficiently inhibited by a variety of the macromolecular crowding agents, such as Ficoll 70, dextran 40, or BSA [224]. Similarly, the recognition of receptor molecules on the host cell membrane by FMDV and infectivity of this virus was efficiently inhibited by short peptides A15 and A19 in dilute solutions, but the inhibition process was noticeably diminished in media containing high concentrations of Ficoll 70 in a crowder concentration-dependent manner [224]. These observations were explained based on the excluded volume theory which, for the association process in crowded environment, predicts strong dependence of the equilibrium association constants on the relative dimensions of interacting molecules [28]. Here, if *I* is the competitive inhibitor and *M* are the competed molecules that can self-oligomerize, and if the sizes of *I* and *M* are similar, then the inhibitory activity of *I* in a crowded solution would be similar to that in dilute solutions. However, if *I* is significantly smaller than *M*, then the inhibitory activity of *I* in crowded medium would be significantly reduced, relative to that in a dilute solution [28]. This is exactly what was observed by the authors analyzing the effect of macromolecular crowding on short peptide-based inhibition of HIV-1 capsid assembly and recognition between the FMDV and FMDV receptors of the host cells [224].

In a similar way, macromolecular crowding with dextran 70 dramatically accelerated polymerization of deoxyhemoglobin S; *i.e.*, promoted the pathogenic process associated with red cell sickling [203]. Although inhibition of this polymerization by non-deoxyhemoglobin S admixtures (such as hemoglobins A, C, and F) was prominent in crowded and non-crowded conditions, the relative efficiency of this inhibition process was not affected much by crowding agents [203].

### 2.7. Phase Separation and Compartmentalization in a Crowded Environment

The space inside the cell is not only crowded, but highly inhomogeneous and compartmentalized, and the cytoplasm and nucleoplasm of any cell contains various membrane-less organelles [225], which are the dynamic assemblies formed via colocalization of molecules at high concentrations within a small cellular micro-domain. Examples of such organelles include promyelocytic leukemia (PML) bodies or nuclear dots, or nuclear domains [226], perinucleolar compartment (PNC) [227], the Sam68 nuclear body (SNB) [227], paraspekles [228], nuclear speckles or interchromatin granule clusters [229], nucleoli [230], processing bodies [231], germline P granules [232,233], Cajal bodies (CBs; [234]), centrosomes [235], and stress granules [236]. Being devoid of membrane, these organelles or bodies are highly dynamic, and their components exist in direct contact with the surrounding nucleoplasm or cytoplasm [237,238]. Many of these structures were shown to be slightly denser than the rest of the nucleoplasm or cytoplasm [239,240], and, despite being considered as a different “state” of cytoplasm or nucleoplasm, they possess biophysical properties similar to those of the rest of the intracellular fluid [225].

These cellular bodies are considered as liquid-droplet phases of the nucleoplasm/cytoplasm [232,236,241,242,243,244] formed via the intracellular phase transitions [225]. These phase transitions in aqueous media originate from the different effects of macromolecules on the structure and solvent properties of water and are related to the high concentrations of macromolecular solutes. At low concentrations of macromolecules, the solution exists as a single phase, whereas at high concentrations, phase separation occurs [245]. Therefore, such phase separation in cytoplasm and nucleoplasm defines the aforementioned compartmentalization and creates a medium with the physicochemical properties similar to those of aqueous two-phase systems [246,247]. For phase separation to occur, the concentration of neutral polymers should be greater than a few percent of each species [248,249]. Protein–protein two-phase systems generally do not form until the concentration of each reaches 7%–10% [250]. These concentrations are high and seldom achieved by single macromolecular species in cytoplasm [246]. The situation is “fixed” by macromolecular crowding, where volume available to any single macromolecule is reduced due to the volume from which it is excluded by surrounding macromolecules in solution thereby leading to the noticeable increase in the local concentration of solutes [246]. In other words, macromolecular crowding represents an important background that affects phase separation of pairs of species, which, in the absence of inert crowders, are not at sufficiently high concentration to separate [251]. In agreement with this notion, calculation based on the Flory-Huggins treatment of concentrated polymer solutions suggested that the presence of 20 wt % of a high molecular weight crowding agent greatly reduces the concentrations needed to produce phase separation [251]. In other words, the requirements for multiphase separation in the liquid phase of cytoplasm or nucleoplasm are met due to the combined effect of high concentration and diversity of some proteins and macromolecular crowding [247,252,253,254].

Crowding was shown to enhance condensation of bacterial DNA by promoting specific interaction of DNA-binding proteins isolated from exponential and stationary phase cell extracts of *E. coli* with the DNA [255]. Importantly, this simple crowding-based mechanism of enhanced protein–DNA interaction provides a reasonable explanation for the fact that cellular DNA in bacteria is maintained in a condensed state, being localized into nucleoids, which are compact, characteristically shaped bodies enclosed by cytoplasm [256]. In fact, digestion of the nucleoid DNA was accompanied by the release of three small proteins (H-NS, FIS, and HU) and RNA polymerase [257], whereas treatment of nucleoids with urea or trypsin converted these compact bodies to partially expanded forms, and treatment with urea was accompanied by the release of most DNA-associated proteins [258].

The optical and electron microscopy-based analyses of the behavior of chromosomes released from mitotic CHO cells (where chromatin is compacted to a concentration of several hundred mg/mL) in solutions containing one of the inert macromolecular crowding agents, such as PEG 8000, dextran 10, or Ficoll 70, clearly showed that the characteristic structure and compaction of these chromosomes were conserved in a crowded environment, and volumes of these chromosomes varied inversely with the concentration of a crowding macromolecule [259]. Curiously, this was in a strict contrast to the inability of other *in vitro* conditions to reproduce the same degree of chromatic compaction. Therefore, macromolecular crowding was proposed to represent one of the major factors defining the compaction of chromosomes [259].

When crowding agents were added to the extracellular culture media containing human mesenchymal stem cells (MSCs), supramolecular assembly and alignment of extracellular matrix proteins deposited by cells was dramatically enhanced [260]. This crowding-directed organization of the extracellular matrix was paralleled by the increased alignment of the intracellular actin cytoskeleton [260]. Another important consequence of this crowding-induced extracellular matrix organization was dramatic change in the adhesion, proliferation, and migration behavior of MSCs [260].

### 2.8. Pathogenic Protein Aggregation and Fibrillation in a Crowded Environment

A number of human diseases, such as Alzheimer, Parkinson, and Creutzfeldt–Jakob diseases are associated with the specific self-association of proteins to amyloid fibrils [261]. These and other protein deposition diseases can be sporadic (85%), hereditary (10%) or even transmissible, as in the case of prion diseases (5%) [262]. Although these diseases are very different clinically, they share similar molecular mechanisms where a specific protein or protein fragment changes from its natural soluble form into insoluble fibrils. Prior to fibrillation, amyloidogenic polypeptides may be rich in β-sheet, α-helix, β-helix, or contain both α-helices and β-sheets. They may be globular proteins with rigid 3D-structure or IDPs, or hybrid proteins possessing ordered and disordered domains [263]. Molecular mechanisms of fibrillation of IDPs and ordered proteins are different [263]: in ordered proteins, the first critical step in fibrillogenesis is the partial unfolding [263,264,265,266,267,268,269,270,271,272], whereas the earliest stage of fibrillation of IDPs is their partial folding [263].

The fibrillation process *in vitro* involves slow nucleation coupled to self-association steps, which constitutes an alternate folding pathway to those leading to the native state [273,274]. Amyloid formation is promoted by destabilization of the native state through events such as mutation or truncation. An illustrative example of a well-studied amyloidogenic protein is α-synuclein, a small (14 kDa) highly abundant presynaptic protein [275,276,277]. The aggregation of this intrinsically disordered protein [75,82,83,278,279,280] is considered as a critical step in Parkinson’s disease (PD), and several other neurodegenerative disorders [281,282,283,284,285,286,287,288]. The link between the conformational behavior and kinetics of α-synuclein fibrillation has been extensively analyzed *in vitro*. Fibrillation was shown to be dramatically accelerated by partial folding of this extended IDP, suggesting that this partially structured conformation is a key intermediate on the fibril-forming pathway [279]. A number of environmental factors have been shown to induce partial folding of α-synuclein and accelerate fibrillation (summarized in [288,289,290]). These fibril-promoting factors include: low pH or high temperature [279], metals [291,292], pesticides/herbicides [292,293,294], polycations and polyanions [295,296,297], low concentrations of organic solvents [298] and osmolytes, e.g., TMAO [299]. Also, the addition of high concentrations of polymers (proteins, polysaccharides, polyethyleneglycols) dramatically accelerated α-synuclein fibrillation *in vitro* [21]. Curiously, the combination of conditions, which induce partial folding of α-synuclein in dilute solutions (low pH, metals, pesticides, heparin, low concentrations of organic solvents and trimethylamine *N*-oxide (TMAO)) was shown to lead to additional acceleration of α-synuclein fibrillation in crowded environment [300,301,302].

Aggregation of human apolipoprotein C-II (apoC-II) that slowly forms amyloid fibrils in lipid-free solutions at physiological pH and salt concentrations was noticeably accelerated by crowding with dextran 10 [20]. Since this crowding agent did not affect the secondary structure of the protein, morphology of resulting fibrils, or the ability of apoC-II amyloid to bind amyloid-specific fluorescent probes thioflavin T and Congo Red, the authors concluded that an increase in the fractional volume occupancy of aggregating protein caused by macromolecular crowding can nonspecifically accelerate the formation of amyloid fibrils by an amyloidogenic protein [20].

In dilute solutions, formation of amyloid fibrils from β-lactoglobulin requires incubation in 2.0 M GdmHCl at room temperature and pH 7.0 for about 20 days [303]. However, shorter lag time and faster growth of fibrils was achieved when this protein was incubated in the presence of high concentrations of dextran 70 and PEG with molecular weights of 400, 8000 and 20,000 [303]. Dramatic acceleration of the amyloid formation in the presence of macromolecular crowding agents has been also reported for human prion protein and a fragment of hyperphosphorylated human tau protein, Tau-(244–441), whereas fibrillation of nonphosphorylated Tau-(244–441) was only moderately promoted by macromolecular crowding [304].

In general, the tendency of proteins to aggregate into non-functional and potentially cytotoxic forms is greatly enhanced by the high total concentration of macromolecules [305]. One should keep in mind that amyloid-like fibrils are not the only possible outcome of the aggregation of a misfolded protein, and the aggregates formed under *in vitro* and *in vivo* conditions exhibit substantial variations in their structure and morphology. In fact, the products of protein aggregation are very different morphologically, varying from soluble oligomers of different shapes and oligomerization degree, to insoluble structure-less amorphous aggregates, and to highly-ordered amyloid-like fibrils. Also, macromolecular crowding might dramatically enhance the competition between protein folding and aggregation, favoring the latter when folding is relatively slow. The kinetics of protein aggregation can be broken down into several major steps: (1) structural transformation within the monomer leading to the appearance of aggregation-prone partially folded species; (2) nucleation and/or formation of oligomers; and (3) formation and growth fibrils or amorphous aggregates. Two latter steps involve association, and the first one involves a conformational change. Obviously, each of these processes is anticipated to be affected by macromolecular crowding in its own way, making the prediction of the net effect of macromolecular crowding on protein aggregation and fibrillation a difficult task [300].

The idea that macromolecular crowding can modulate the aggregate heterogeneities was also supported by the analysis of the effects of macromolecular crowding on amyloid formation of a model amyloidogenic peptide (RATQIPSYKKLIMY) derived from the *C*-terminal region (amino acids 248–286) of PAP [306]. This analysis revealed that the addition of macromolecular crowding agents affected the morphology of the amyloid fibrils formed by this peptide leading to the formation of shorter amyloid aggregates, thereby supporting the hypothesis that the heterogeneous classes of aggregates can be favored by macromolecular crowding [306].

To support this view, the effect of macromolecular crowding on fibrillation of four proteins, bovine *S*-carboxymethyl-α-lactalbumin (a disordered form of the protein with three out of four disulfide bridges being reduced), human insulin, bovine core histones, and human α-synuclein were analyzed [300]. These proteins of interest were structurally different, varying from extended IDPs (α-synuclein and core histones) to folded proteins with rigid tertiary and quaternary structures (monomeric and hexameric forms of insulin). Although all these proteins were shown to fibrillate, they possess very diverse aggregation mechanisms and, depending on the environmental conditions, were able to form different aggregates in addition to amyloid-like fibrils [300]. The data obtained in this study gave rise to several important conclusions, such as: (a) A crowded environment slows down or inhibits fibrillation of oligomeric proteins irrespectively of the level of structural order in their initial conformation; (b) Fibrillation of monomeric IDPs is accelerated by high concentrations of crowding agents; and (c) If conditions favors multiple folding and aggregation pathways leading to co-existence of different folded and aggregated species, macromolecular crowding works toward the most thermodynamically favorable conformation helping the system to eliminate less favorable ones [300].

In agreement with the idea that various aggregation pathways can be variously modulated by macromolecular crowding, Huang *et al.* showed that under the conditions of macromolecular crowding, the recombinant human prion protein can be converted from a soluble monomeric, α-helical conformation into β-sheet cytotoxic oligomers resistant to proteinase K digestion [200]. An analysis of the self-association of the type-2 diabetes mellitus related human islet amyloid polypeptide (hIAPP) in various crowded environments revealed the existence of two competing processes, where globular off-pathway species were stabilized in a crowder concentration and type-dependent manner and where hIAPP fibrillation reaction was retarded or even inhibited [307]. Curiously, crowding-stabilized globular off-pathway species were shown to be non-toxic in the hIAPP cytotoxicity assays on pancreatic β-cells, where the species formed in the normal fibrillation pathway were characterized by the high cytotoxicity [307].

Recent work of Ma *et al.* provide strong evidence in support of the idea that macromolecular crowding is able to accelerate fibrillation of amyloidogenic proteins, whereas non-amyloidogenic proteins stay mostly non-aggregated even in the presence of high concentrations of crowding agents [308]. In fact, these authors showed that although fragments of phosphorylated human Tau did not form fibrils in the absence of a crowding agent, they did efficiently fibrillate in a crowded environment. The presence of crowding agents also accelerated amyloid fibril formation of human prion protein and its two pathogenic mutants E196K and D178N, as well as of a pathological human SOD1 mutant A4V [308]. On the other hand, non-amyloidogenic proteins, rabbit prion protein and hen lysozyme, failed to form amyloid fibrils even when a crowding agent was present at a concentration of 300 mg/mL [308].

Curiously, the analysis of the effect of synthetic macromolecular crowders (Ficoll 70 and Dextran 70) on the aggregation of a β-rich protein, bovine carbonic anhydrase (BCA), revealed that both Ficoll 70 and Dextran 70 were able to dramatically decrease the extent of BCA aggregate formation [309]. This peculiar behavior was explained in terms of the decrease in the population of aggregation-prone intermediates as a consequence of increased native state stability [309].

### 2.9. Inequality of Crowding Agents

It is recognized now that not all crowders are made equal. This inequality is very protein-specific (*i.e.*, crowders that efficiently act on some proteins would be less efficient on some other proteins), suggesting that there is a clear deviation from the simple excluded volume model, and that in addition to hard non-specific steric interactions some protein-specific “soft” interactions between proteins and crowders might be present.

Comparative study of the enzymatic activity modulating effects of high concentrations of globular proteins (hemoglobin and lysozyme), various dextrans, and PEGs revealed that crowding agents are not equal in their ability to affect the reaction rates of several decarboxylating enzymes, such as urease (a multisubunit enzyme (EC 3.5.1.5)), pyruvate decarboxylase (a homotetrameric enzyme (EC 4.1.1.1)) and glutamate decarboxylase (a monomeric enzyme (EC 4.1.1.15)) [310]. Here, the authors showed that the increase in the concentrations of globular proteins caused a dramatic increase in enzymatic activity with up to 30% crowding concentration, whereas further increase in concentration of protein-crowders resulted in activity decrease. On the other hand, a concentration-dependent decrease in activity was observed in the presence of dextrans and PEGs [310].

Curiously, the analysis of the effects of high concentrations of high molecular weight polymers and their low molecular weight derivatives revealed that only concentrated solutions of large polymers (such as PEG 8000, glycogen, and Ficoll 70) were able not only increase the catalytic efficiency of the T4 polynucleotide kinase [311,312], but also resulted in noticeable stabilization of this kinase and prevented its inactivation [311]. In this case too, the excluded volume was suggested as a mechanism defining the increase in the efficiency of the kinase-DNA binding [311]. Another protein for which high concentrations of high molecular weight macromolecular crowding agents (PEG 6000 and PEG 35,000), but not their lower molecular weight analogue (PEG 1000) were able to dramatically increase the catalytic efficiency was *Escherichia coli* ADP-sugar pyrophosphatase (AspP), which is a “Nudix” hydrolase that catalyzes the hydrolytic breakdown of ADP-glucose linked to glycogen biosynthesis [313].

In agreement with the aforementioned point that not all crowders are made equal, the analysis of oxidative folding of four disulfide bond-containing model peptides of varying molecular size from 13 residues (1.4 kDa) to 58-residues (6.5 kDa) (conotoxins GI, PVIIA, and r11a, and bovine pancreatic trypsin inhibitor) revealed that the addition of high concentrations of polysaccharides did not affect the folding rates nor equilibria for these peptides, whereas folding reactions were dramatically accelerated in the presence protein-based crowding agents (albumin or ovalbumin), even when concentrations of these crowders were lower than those predicted to provide the excluded volume effect [314]. Since both protein-based crowding agents used in that study contained cysteines, it has been concluded that they serve as redox-active crowding agents [314].

Zhang *et al.* showed that crowding agents Ficoll 70, dextran 70, PEG 2000, and PEG 20,000 have different effects on the structural stability of human α-lactalbumin (HLA) and transition of this protein to a molten globule state [315]. It is known that the thermal-induced molten globule state can be induced in Ca^2+^-depleted HLA (apo-HLA) at pH 7.0 and 55 °C (*T*_m_ of this process in dilute media is 39.7 °C), whereas the acid molten globule of this protein is realized at pH 3.0 and 25.0 °C. The thermal stability of apo-HLA was significantly decreased by high concentrations of PEG 2000 and PEG 20,000 (the corresponding *T*_m_ decreased by 6 and 4 °C, respectively) thereby leading to the formation of a stable molten globular state at lower temperatures. On the contrary, high concentrations of dextran 70 and Ficoll 70 dramatically enhanced the thermal stability of apo-HLA (by 12.6 and 3.8 °C, respectively) [315]. The difference in the action of different crowders on thermal stability of apo-HLA was attributed to the fact that no detectable interaction was observed between apo-HLA and Ficoll 70 or dextran 70, whereas there was a weak, non-specific interaction between the protein and PEG 2000 [315].

Similarly, the unfolding behavior of myoglobin (Mb) was differently affected by Ficoll 70, dextran 70, and dextran 40 [316]. Here, the analysis of thermal denaturation suggested that all the crowders at the concentration of 200 g/L had a destabilizing effect on Mb as compared to the thermal unfolding of this protein in dilute solutions [316]. However, when Mb subjected to thermal denaturation was originally destabilized with different urea concentrations, Ficoll 70 showed a destabilizing effect on the Mb unfolding transition under all conditions, whereas dextran 40 and dextran 70 were destabilizing at 0–2 M urea but did not have such additional destabilizing effects when thermal denaturation was monitored in the presence of 3–5 M urea [316]. Ficoll 70 and Dextran 70 were also shown to differently affect the aggregation propensity of a β-rich protein BCA [309]. Here, the extent of BCA aggregation was noticeably lower in the presence of Dextran 70 relative to that in the presence of Ficoll 70 (for the same *w*/*v*).

Most studies of the effects of crowding on protein structure and aggregation have used flexible, hydrophilic polymers (PEG, dextran and Ficoll) [317]. Due to their compact, largely spherical shape these polymers have relatively small surface to volume ratio. They are neutral and relatively hydrophilic minimizing their specific interactions with proteins. Thus these polymers are believed to act primarily via excluded volume effect by decreasing the effective volume available for the proteins in the cell and thus increasing the effective protein concentration. However, a variety of other, more rigid biopolymers are also present *in vivo* including DNA, protein fibers and polysaccharide components of extracellular matrix. Solutions of rigid polymers have higher viscosity that may affect protein diffusion and slow down protein folding or aggregation. These polymers also have lower density making them more effective in creating excluded volume effect as intrinsic viscosity of a polymer is proportional to its volume [318]. Lower polymer density also increases the exposed surface of rigid polymers available for interactions with proteins. To understand the effect of the crowder rigidity on its ability to affect protein structure and aggregation, the effects of both compact, flexible polysaccharides (dextrans) and more rigid cellulose derivatives (hydroxypropyl celluloses or HPCs) were studied on the kinetics and mechanism of aggregation of several proteins with variable degrees of intrinsic disorder and oligomeric state [317]. In this study, neutral, hydrophilic polysaccharides were selected to minimize the specific protein-polymer interactions. Since these polymers have the same charge and hydrophobicity, they presented a good test case of the effect of shape and structural rigidity of polymers on protein aggregation. The analysis revealed that in the presence of dextrans fibril formation from the proteins that are either present as stable oligomers (insulin, pH 7.5) or form oligomers in the process of aggregation (α-lactalbumin) was inhibited. On the other hand, fibril formation by IDPs (histones and α-synuclein) was accelerated. Effect on monomeric folded proteins (lysozyme and insulin at pH 2.5) was intermediate with either weak acceleration or weak inhibition [317]. HPCs inhibited fibril formation from all proteins except histones. All aggregation parameters were affected with significant increases in the lag phase and decreases in ThT fluorescence and the fibril elongation rate. Indifference of histones to the presence of HPCs may be due to the inability of histones to partially fold in the assay conditions [317]. Data reported in this study suggest that biopolymers influence protein aggregation via a complex interplay of several mechanisms including excluded volume effect, solution viscosity, and protein-polymer interaction [317].

A recent analysis of the effect of various crowding agents on interaction between the Ca^2+^/CaM-dependent protein kinase II (CaMKII) and the NMDA-type glutamate receptor (NMDAR) subunit GluN2B revealed that the Ca^2+^/CaM-regulated formation of the CaMKII-GluN2B complex is differently affected by different macromolecular crowders [319]. The authors showed that this interaction was reduced by lysozyme but enhanced by BSA. Similar to BSA, high concentrations of dextran 10, dextran 70 and PVP 40 resulted in the more efficient formation of the CaMKII-GluN2B complex, whereas the addition of 100 mg/mL of non-specific rabbit IgG reduced CaMKII binding to GluN2B [319]. The inhibitory effects of lysozyme and IgG in the presence of Ca^2+^ were attributed to the specific or non-specific binding of the regulatory CaM protein to these protein crowders [319].

### 2.10. Effects of Mixed Macromolecular Crowding Agents

Another illustration of the inequality of crowding agents is given by the observation that mixed macromolecular crowding agents affect protein behavior differently than solution of the individual crowders.

The use of a mixed macromolecular crowding agent containing both BSA and polysaccharide resulted in a remarkable increase in the yield of oxidative refolding of reduced, denatured hen egg white lysozyme, suggesting that the mixed crowded media could be more favorable for protein folding [172]. In agreement with this hypothesis, four single macromolecular crowding agents, Ficoll 70, dextran 70, PEG 2000, and calf thymus DNA (CT DNA), and three mixed crowding agents containing both CT DNA and polysaccharide (or PEG 2000) differently affected the refolding of guanidine hydrochloride-unfolded rabbit muscle creatine kinase (MM-CK) [320]. Here, mixed crowded media were more favorable for MM-CK refolding and induced noticeably less aggregation of this protein than the individual crowding agents by themselves [320]. It was also pointed out that the mixed crowding media containing two crowding agents, Ficoll and dextran, simultaneously was able to exert a greater stabilizing effect than the sum of the two individual crowding agents, suggesting that the composition of crowders, not just their total concentrations, might play an important role in the ability of the crowded environment to modulate protein folding [321]. Rather similar data were also reported for the refolding and aggregation of the GdmHCL-unfolded recombinant human brain-type creatine kinase (rHBCK) in the presence dextran 70, PEG 2000, and CT DNA and different mixtures of these crowding agents [322].

Besides being important for regulation of protein refolding, the mixed crowded environment containing both BSA and Ficoll 70, but not Ficoll 70 alone, was shown to almost completely inhibit amyloid formation by hen egg white lysozyme and stabilize the native form of this protein [323].

## 3. Conclusions

This review provides compelling evidence that macromolecular crowding represents a very important factor that affects various aspects of protein life. Obviously, due to its immense biological importance macromolecular crowding cannot be ignored. It is almost impossible to find a single protein-related feature that would not be affected by the addition of high concentrations of macromolecules. In fact, biological activities, structural features of ordered and intrinsically disordered proteins, shape of protein molecules, protein dynamics, protein self-assembly, assembly and ability to interact with various partners or to form functional or pathogenic complexes, protein oligomerization, aggregation, and fibrillation, protein folding, structural stability, and conformational behavior, phase separation and compartmentalization, as well as other aspects of these important building blocks of life can be tuned by the extremely crowded cellular milieu or by the concentrated solutions of various synthetic and biological polymers that serve as model “crowding agents” in typical *in vitro* experiments.
